# Virgin Coconut Oil and Its Lauric Acid, Between Anticancer Activity and Modulation of Chemotherapy Toxicity: A Review

**DOI:** 10.3390/jox15040126

**Published:** 2025-08-05

**Authors:** Debalina Bose, Adetayo Olorunlana, Rania Abdel-Latif, Ademola C. Famurewa, Eman M. Othman

**Affiliations:** 1 Advanced Technology Development Centre, Indian Institute of Technology Kharagpur, Kharagpur 721302, India; debalinabose22@gmail.com; 2 Department of Nursing, College of Nursing and Basic Medical Sciences, Caleb University, Lagos 100211, Nigeria; adetayo.olorunlana@calebuniversity.edu.ng; 3 Department of Pharmacology and Toxicology, Faculty of Pharmacy, Minia University, Minia 61519, Egypt; rania.abdellatief@mu.edu.eg; 4 Department of Medical Biochemistry, Faculty of Basic Medical Sciences, College of Medical Sciences, Alex Ekwueme Federal University, Ndufu-Alike Ikwo, Abakaliki 482131, Nigeria; 5 Centre for Natural Products Discovery, School of Pharmacy and Biomolecular Sciences, Faculty of Science, Liverpool John Moores University, Byrom Street, Liverpool L3 3AF, UK; 6 Department of Biochemistry, Faculty of Pharmacy, Minia University, Minia 61519, Egypt; 7 Cancer Therapy Research Center (CTRC), Department of Biochemistry-I, Biocenter, University of Wuerzburg, Theodor-Boveri-Weg 1, 97074 Wuerzburg, Germany

**Keywords:** virgin coconut oil, lauric acid, anticancer, chemotherapy toxicity, apoptosis

## Abstract

Virgin coconut oil (VCO) has emerged as a functional food oil with considerable health benefits and wide applications in the food, pharmaceutical, and cosmetic industries due to its resident bioactive compounds, including lauric acid (LA). LA is the most abundant saturated medium-chain fatty acid in VCO and has been associated with several pharmacological activities. The literatures show the pharmacological effects of VCO and LA on chronic pathologies, infectious diseases, and metabolic disorders. A robust body of evidence shows that LA and other phenolic compounds are responsible for the VCO protection against toxicities and pharmacological efficacies. This review elucidates the anticancer mechanisms of VCO/LA and their modulation of the chemotherapy-induced side effect toxicity. VCO, LA, and their nanomaterial/encapsulated derivatives promote ROS generation, antiproliferation, apoptosis, cell cycle arrest, the inhibition of metastasis, and the modulation of cancer-related signaling pathways for cancer cell death in vivo and in vitro. VCO mitigates oxidative inflammation and apoptosis to block the underlying mechanisms of the side effect toxicity of chemotherapy. However, the possible beneficial effect of LA on the toxicity of chemotherapy is currently unknown. The available evidence emphasizes the anticancer effect and mechanism of VCO and LA, and the VCO potential to combat adverse side effects of chemotherapy. Thus, VCO and LA are potential adjuvant therapeutic agents in the management of various cancers. Nevertheless, future studies should be targeted at elucidating cancer-related molecular mechanisms to bridge the gap in knowledge.

## 1. Introduction

Cancer continues to pose a significant global health challenge with increasing prevalence despite the Food and Drug Administration (FDA) approval of more than 600 cytotoxic anticancer agents [1]. Chemotherapy is the clinical cornerstone for metastatic cancer treatment globally. It is acknowledged for systemic efficacy, patient improvement, and survival; however, it is increasingly confronted with severe side effects, chemoresistance, and relapses [2,3]. These formidable hurdles hamper clinical chemotherapy effectiveness; therefore, there is an unmet need for alternative therapies, adjuvants, or combination regimens to combat the growing health problem [4]. In this pursuit, there is a growing research interest in exploring the potential therapeutic benefits of natural products for cancer treatment. The multi-target novel adjuvants or treatment agents based on natural products with higher anticancer potency and low toxicity profiles are required for the treatment of cancers [4,5]. Some plant-derived natural products have shown potential anticancer potential in combination therapies, though safety and efficacy must be assessed case by case [6]. Among these potential products is VCO gradually emerging as a potential candidate due to its potential anticancer properties and ability to mitigate the toxicity associated with chemotherapy [7,8]. VCO is extracted from the kernel or meat of mature coconutsand has been recognized for its diverse health benefits [9]. There is a growing demand for VCO in the United Sates and other developed countries, and this demand can be attributed to the increasing number of reports and scientific literature published on the health benefits of VCO [10]. The quantity of VCO exported by India, Philippines, Indonesia, West Malaysia, Sri Lanka, Mexico, and Papua New Guinea, the major countries involved in the large production of VCO, has increased over the years [10,11,12]. VCO is a natural extract of the fresh, mature kernel (meat) of coconuts without the use of heat, without undergoing chemical, refining, bleaching, or deodorizing processes [10]. The literature has shown the analgesic, immunomodulatory, antipyretic, antioxidant, anti-stress, antimicrobial, anti-HIV, antidiabetic, antiviral, anti-inflammatory, anti-obesity, hypocholesterolemia, and anticancer effects of VCO [11,13,14]. A number of studies have also demonstrated the VCO capacity to inhibit the toxicity induced by clinical drugs and various chemicals [15,16,17,18]. In particular, the anticancer drugs used in chemotherapy induce various side effect toxicities in cancer patients [19,20]. Preclinical evidence reveals that it exerts toxicity on the liver, kidney, heart, testis, placenta, brain, lung, ovary, intestine, blood, and thyroid gland [19,20,21,22]. The underlying mechanisms of toxicity strongly reported by experimental studies include oxidative stress, pro-inflammation, apoptosis, and mitochondrial dysfunction [23]. But a number of findings in animal studies indicate that VCO may exert beneficial effects against the toxicity of chemotherapeutic drugs [22,23,24,25]. The beneficial effects are attributed to its unique composition of medium-chain triglycerides (MCTs), medium-chain fatty acids (MCFAs), phenolic compounds, and other bioactive molecules [11,26].

Notably, it is LA that constitutes a substantial portion of the VCO fatty acid profile, and there is a renewed focus on LA in cancer research circles. LA, the most abundant MCFA in VCO, has demonstrated synergistic effects when combined with conventional anticancer drugs; it showcases selective cytotoxicity against cancer cells while sparing normal cells [27,28,29]. Mechanistic investigations have unveiled its capacity to induce apoptosis, inhibit cancer cell proliferation, and interfere with various signaling pathways involved in cancer development and progression [27,30]. Moreover, while the research on VCO’s anticancer effects is still growing, the available evidence suggests potential for this natural oil in cancer prevention and management. By examining the molecular mechanisms of action and evaluating their efficacy in preclinical and clinical settings, a deeper understanding of how VCO and its constituents may complement current cancer treatment regimens can be attained. Additionally, exploring their role in mitigating the toxicity of anticancer drugs offers valuable insights into their potential as adjunctive therapies in cancer management. This ongoing exploration holds the promise of contributing to advancements in oncology research and clinical practice, paving the way for improved cancer treatment and prevention strategies.

## 2. Extraction and Composition of VCO

VCO is an unprocessed oil extracted from the mature and fresh white kernel of coconut fruit, Cocos nucifera L. VCO is obtained from fresh, mature coconuts by mechanical or natural means without refining, chemicals, preservatives, or high heat [31,32]. The method preserves its nature, color, distinct flavor, and aroma, maintaining marginal differences in the iodine value, saponification value, refractive index, fatty acid profile, specific gravity, and moisture content compared to copra coconut oil extracted under a high temperature ranging from 204OC to 245OC [32]. At a low temperature of about 30OC, VCO is colorless but appears white at a lower temperature and solid form. It is majorly made up of saturated fats (about 94%), and medium-chain fatty acids (above 62%) of which about 45–52% is LA [11,33]. Traditionally, there are two types of coconut oil, the VCO and the copra coconut oil, based on the method adopted (wet or dry process) during extraction from the coconut kernel meat.

### 2.1. Extraction of VCO (Wet Process)

**Figure 1:**
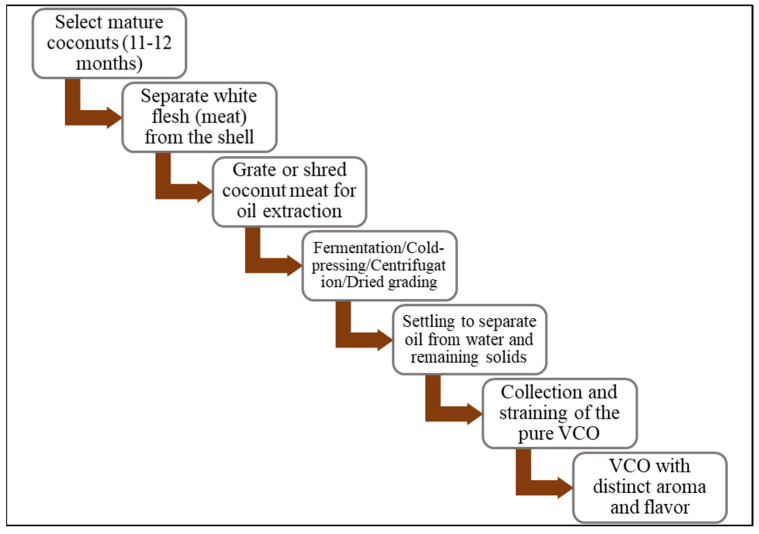
A schematic process flowchart for the extraction of VCO.

**Figure 2:**
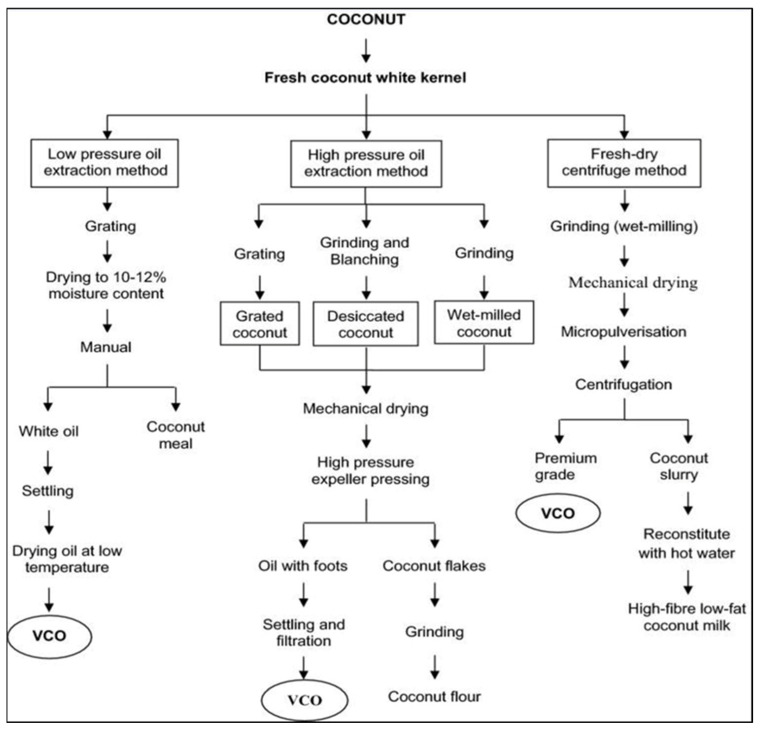
A schematic flowchart of methods and processing stages for VCO production.

VCO is obtained by the mechanical extraction of coconut kernel without refining or high-temperature treatment, preserving its native properties [34]. Various sub-methods of the wet extraction process exist (Figure 1) in which the production of VCO occurs without RBD, thus preserving the contents and nature of the oil [31,35]. The wet process typically begins with the use of mature coconuts known for their high oil content. The kernel is grated or shredded to facilitate oil extraction. This is followed by either cold-pressing—where mechanical pressure is applied at low temperature or centrifugation, which separates the oil from coconut milk through high-speed spinning. Both methods avoid high heat and chemical processing, preserving the natural composition of the oil, including its lauric acid content. The extracted oil is then clarified through settling and filtration to remove moisture and impurities, resulting in pure VCO (Figure 2). One of the key advantages of natural VCO preparation is the retention of its nutritional integrity. VCO obtained through natural wet methods retains its beneficial fatty acids, medium-chain triglycerides (MCTs), antioxidants, and other micronutrients. This makes natural VCO a preferred choice for those seeking a wholesome, unadulterated source of coconut oil for culinary and wellness purposes. Furthermore, the natural preparation of VCO aligns with sustainable and environmentally friendly practices, as it minimizes the use of chemicals and energy-intensive processes. By exploring the inherent properties of coconuts and employing traditional extraction methods, producers can create a pure and authentic product that embodies the essence of VCO [34]. Other techniques for the optimal removal of the oil from the coconut milk emulsion include enzyme-assisted extraction [36,37,38], chilling, freezing, and thawing techniques [35], and fermentation, a traditional method wherein coconut milk is extracted, allowed to settle, and undergoes natural fermentation for 24–36 h within which the oil phase becomes separated from the aqueous phase. The further slight heating of the oil phase for a short time removes the moisture, leading to oil separation [12,21]. The main demerits of this process are the low oil recovery and fermented odor of VCO. Bawalan [39] detailed this process under wet processing, where the separated oil is filtered and collected as VCO. Likewise, Srivastava et al. [40] employed wet processing with cold extraction, where the coconut milk was left to settle under controlled conditions (35–40 °C, 75% humidity) for 20–24 h. The airborne lactic acid bacteria help in breaking protein bonds, aiding in VCO separation. However, although the method is attractive and produces quality oil, it yields a comparatively low amount of oil, which has discouraged its commercial application.

### 2.2. Extraction of Copra Coconut Oil (Dry Process)

Traditionally, coconut oil is extracted by pressing copra (dry process) that has been exposed to a very high temperature and/or sunlight to remove moisture [41]. The coconut oil is produced by crushing copra, which contains about 60–65% of the oil [12]. Copra is the dried kernel obtained by smoke-drying, sun-drying, or a combination of both. Then, the clean, ground, and steamed copra is pressed with a screw press expeller or hydraulic press to obtain the copra coconut oil. However, the drying/copra method is traditionally unhygienic; in fact, the handling makes the extracted oil unsafe for human consumption. Therefore, the coconut oil undergoes a refining, bleaching, and deodorizing process. This process requires heating the oil at high temperatures, between 204 °C and 245 °C, which destroys the essential amino acids, tocopherols (e.g., vitamin E), and other valuable phytocompounds present in the coconut oil [10]. Hence, VCO is a healthier oil than commercial copra oil due to its medium-chain saturated fatty acid content and higher amounts of polyphenols [11,42].

### 2.3. Composition of VCO

**Figure 3:**
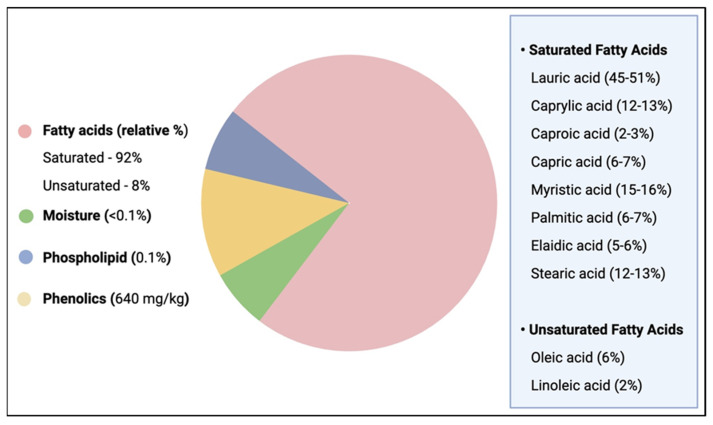
Composition and percentage component of VCO.

VCO is the pure and healthy form of coconut oil with a coconut aroma and a clear water appearance in contrast to copra coconut oil with a yellow color, which is sometimes due to microbial contaminants or high-temperature processing [10,43]. VCO is rich in natural bioactive compounds including saturated fatty acids (SFAs) (mainly medium-chain FAs), and phospholipids (e.g., tocopherols and sterols), as well as phenolic acids and polyphenols, in addition to minor components like flavonoids [40] (Figure 3).

These bioactive constituents contribute to a variety of biological effects, including antioxidants, anti-inflammatory, antimicrobial, and anticholesterolemic effects (Table 1).

Due to the high content of SFAs, some authors have reported that its continued consumption may increase low-density lipoprotein (LDL) [50,51]. However, unlike the long-chain fatty acids, the physicochemical properties of VCO enhance its intestinal absorption via the hepatic portal vein to hepatic metabolism for the production of energy [41,52] and does not essentially contribute to cholesterol synthesis. Moreover, the medium-chain triglyceride derivatives of MCFAs are bioactive forms with versatile pharmacological properties that can help in boosting the immune system, treating various pathologies such as cardiovascular, gastrointestinal, and inflammatory disorders. They have the capability to fight off numerous bacterial, fungal, and viral infections [53]. In human, the oral ingestion of VCO stimulates the conversion of LA, the most abundant MCFA (45 to 52%), to monolaurin, a key component of breast milk that boosts the baby immune system and has the potential to damage bacteria’s lipid membranes.

Moreover, studies demonstrate that VCO contains nearly seven times more total phenolic content compared to regular coconut oil, underscoring its superior antioxidant capacity and health benefits. This difference arises because the refining process of commercial coconut oil can diminish its biologically active components [54]. While the exact composition of phenolic acids in VCO may vary depending on factors such as the coconut variety, growing conditions, and extraction methods, several phenolic acids are commonly found in this oil.

## 3. Health Benefits of VCO and LA

### 3.1. VCO Health Benefits

Functional foods are food substances that provide health benefits beyond basic nutrition and that are capable of preventing diseases (Temple, 2022). VCO is a rapidly emerging functional food oil whose popularity and public awareness is increasing worldwide [35]. It is used in food industries for different purposes; it is an integral part of Sri Lankan and many South Asian diets. Being rich in MCFAs and highly digestible, VCO is increasingly available, especially in Southeast Asia. Its benefits include antioxidant activity and various health advantages, thus making it a desirable addition to culinary practices. Recent research findings support its physicochemical properties and clinical benefits, further boosting its appeal in the food industry [35]. Nevertheless, the use of coconut oil in one’s diet remains controversial owing to its high SFA content. In decades past, high SFA in one’s diet was associated with elevated total cholesterol and low-density lipoprotein (LDL) levels in blood circulation leading to dyslipidemia and cardiovascular diseases (CVDs) [44]. Coconut oil has received an unfavorable reputation because there are claims that it may enhance cholesterol synthesis, and hypercholesterolemia leading to CVDs. However, in the past few years, clinical and non-clinical studies have been conducted on coconut oil and VCO, and positive outcomes were obtained, which might refute those arguments [41,44,55]. The MCFAs abundant in VCO have different chemical and physiological properties when compared to long-chain fatty acids (LCFAs). In human cells, MCFAs and LCFAs are metabolized differently by different lipase enzymes [56,57]. MCTs are hydrolyzed by lingual (mouth) and gastric (stomach) lipases into 2-monoacylglycerol and fatty acids, mostly MCFAs, which are rapidly absorbed by the enterocytes into the portal vein and then directly enter the liver to be quickly metabolized into energy; hence, MCTs do not increase blood triglycerides or LDL levels [56,58]. Additionally, MCTs do not require bile salts for digestion, making them more rapidly absorbed [59,60,61] whereas the pancreatic lipases digest the dietary long-chain triglycerides into free long-chain fatty acids and then become absorbed by the enterocytes and resynthesized into new triglycerides. The new triglycerides enter the lymph system as chylomicrons, and are then transported into the heart and circulation, hence, contributing to blood long-chain triglycerides and the synthesis of LDL. Therefore, VCO does not increase LDL levels, but increases HDL levels and decreases the LDL/HDL ratio and CVD susceptibility [56,58]. However, there are inconsistent reports often arising from different sources and methods of coconut oil production, population differences, and small trial participant size [50,62]. For example, a review on coconut oil and cardiovascular risk factors in humans concluded that the evidence of an association between coconut oil consumption and blood lipids or cardiovascular risk was mostly of poor quality [51]. Interestingly, a randomized clinical trial that compared the effects of VCO (saturated fat), butter (saturated fat), or virgin olive oil (unsaturated fat) on the blood lipid profile shows that the effect of VCO is comparable to that of virgin olive oil, whereas butter significantly increased the LDL-C concentrations [50] The study indicates that VCO significantly increased HDL-C and had a similar effect on the TC/HDL-C ratio as olive oil compared with butter. In another recent randomized trial [63] conducted in the UK, VCO and virgin olive oil had similar beneficial health effects on circulating odd-chain saturated fatty acids and trans-fatty acids by reducing their levels, while butter increased the levels of these fatty acids.

Compared to butter and hydrogenated oils, VCO has a higher content of medium-chain triglycerides, lacks cholesterol and trans fats, and demonstrates faster metabolic oxidation. These distinctions may underline some of the health-related claims attributed to VCO, particularly in the context of lipid metabolism and cardiovascular outcomes (Table 2).

Being rich in MCFAs, VCO prevents infections and promotes health by lowering cholesterol through thyroid stimulation. Its high resistance to oxidative rancidity makes it ideal for cooking [64]. It serves as an excellent carrier for fat-soluble vitamins and has notable antimicrobial properties [64]. VCO is renowned for promoting cholesterol reduction, cardiovascular health, weight management, cognitive function, and antimicrobial effects [65]. VCO has gained popularity for its potential health benefits, especially in cardiovascular health. According to a study by Dumancas et al. (2016) [10], VCO shows comparable effects to other saturated fats on LDL levels but may increase HDL levels. Its antioxidant property suggests potential cardiovascular benefits by reducing oxidative stress, though further research is needed. Research comparing extra virgin coconut oil (EVCO) with extra virgin olive oil (EVOO) indicates that EVCO may be more effective in reducing hunger and the desire to eat, particularly in normal-weight individuals [66]. VCO can aid in weight loss by enhancing satiety and increasing thermogenesis, which promotes fat loss directly from adipose tissue [67]. Its phenolic compounds also contribute to reducing the risk of atherosclerosis by decreasing lipid peroxidation and normalizing lipid levels [65].

Moreover, VCO exhibits antidiabetic, anti-obesity, and antiasthmatic properties by enhancing insulin secretion, and inhibiting fat accumulation, alongside potent antimicrobial activity against diverse pathogens, including bacteria, viruses, fungi, and protozoa [13,68,69,70,71,72,73,74,75,76]. VCO has been shown to be beneficial against ulcerative colitis, experimental infertility, inflammatory bowel disease, antibiotic gentamicin toxicity, and oral cancer [77,78]. VCO increases the absorption of calcium and magnesium, compared to other types of oils with a high content of long-chain fatty acids; it prevents obesity, and, hence, decreases the incidence of or prevents diabetes, and induces insulin sensitivity [56]. The combined topical application of VCO and black cumin oil enhances diabetic wound healing through the increased expression of the vascular endothelial growth factor (VEGF) gene, an essential mediator of angiogenesis and tissue regeneration [79]. The beneficial health effects of VCO in polycystic ovarian syndrome and metabolic syndrome have been reported in the existing literature [80,81]. In pharmaceutical settings, VCO shows promise as a carrier for lipid-soluble drugs when processed under controlled conditions [82]. VCO is being explored for its potential in Alzheimer’s disease (AD) treatment, particularly through formulations designed to increase brain cholesterol levels and mitigate β-amyloid plaque formation, which are characteristics of AD [83]. Aluminum-induced AD-like neurological damage and neurobehavioral dysfunction were alleviated by oral VCO [84]. Aluminum-induced neuroinflammation and oxidative toxicity were suppressed via the regulation of the NLRP3 inflammasome triggered by betaine in combination with VCO [85]. Moreover, a review of the anti-Parkinson-disease effects of VCO is chronicled by Deepika et al. (2024) [86], while the health effects of VCO in enhancing memory and neurobehavioral activity were shown by the study of Rahim et al. (2017) [87] and de Vasconcelos et al. (2023) [88] respectively. These studies highlight VCO’s emerging role in neurological health. The anticancer effects of VCO and its modulation against toxicities of various environmental pollutants or toxicants have been reported [89,90,91,92,93,94,95]. The anticancer effects of VCO have been reported in the literature for a number of cancer cell lines [89]. Both in silico and in vitro analyses show that VCO may inhibit certain hallmarks of cancer development [96,97]. Experimental arthritis induced by an intradermal injection of complete Freund’s adjuvant was suppressed by VCO polyphenolics via the modulation of the antioxidant and anti-inflammatory COX-2/iNOS/TNF-α/IL-6 pathway [98]. Moreover, the anti-HIV/AIDS effect of VCO was also demonstrated in a clinical case–control study that enrolled HIV-positive subjects with a CD4+ T lymphocyte count > 200 cell/µL [99]. In the study, it was found that the VCO supplementation significantly increased the CD4+ T lymphocyte count. In another clinical study on COVID-19-positive patients in a 28-day randomized, single-blind trial, adjunct VCO therapy was found to alleviate symptoms and suppress the C-reactive protein concentration [100]. Clinical studies underscore its ability to alleviate skin disorders through its moisturizing and soothing effects, devoid of skin irritants and phototoxicity, alongside potent anti-inflammatory properties that bolster overall skin health [101,102]. Overall, VCO’s unique FA composition distinguishes it from other oils and contributes to its beneficial effects on body fat reduction, heart health, hyperglycemia, and metabolic disease prevention, and potential antibacterial actions which support its broader role in enhancing overall health [103]. These attributes collectively position VCO as a versatile and potential candidate for further research and development across various healthcare applications.

### 3.2. LA Health Benefits

LA is a 12-carbon medium-chain saturated fatty acid found abundantly in coconut oil or palm kernel oil, recognized to play a vital role in pathologies and improving immune physiological function, and it comes with multiple beneficial effects [14,104]. Alves et al. (2017) [105] found that LA lowers the experimental blood pressure and oxidative stress in rats. Reports show that LA may modulate genes that underline atherosclerotic plaque formation [106]. In the THP-1 macrophage model, LA ameliorates the insulin resistance via the upregulation of the glucose uptake, mitochondrial membrane potential, ATP generation, and expression of mitochondrial biogenesis regulator genes such as GLUT-1, GLUT-3, TFAM, PGC-1α, and PPAR-γ [14]. In comparative studies, LA exerts more health effects compared to palmitic acid with respect to blood glucose reduction, insulin resistance, metabolic inflammation, and mitochondrial health in human primary myotubes and animal models [104,107,108]. It exhibits significant antimicrobial and antioxidant properties, making it valuable in both nutritional and pharmacological applications [56]. Its effectiveness against microbial growth and oxidative stress enhances its role in promoting health and treating skin conditions. LA improves the antioxidant and immune capacity for the intestinal structure while reducing the microbial population of aquatic organisms [109]. Although this study reveals an immunomodulatory effect of LA in the intestine of swimming crabs, the effect of LA on immunity in animal models should be investigated. LA also shows beneficial health effects by stabilizing the immune status in European Seabass Juvenile fish [110]. LA as monolaurin demonstrates a potential anti-retroviral effect via reductions in the viral load with variable changes in CD4 counts, and shields the body from parasites [33,111]. The antioxidant, anti-inflammatory, and anti-apoptosis mechanisms of LA have been demonstrated in the literature. A recent study reveals that LA modulates ethanol-induced hepatotoxicity via the suppression of hepatic oxidative stress, cytokine inflammation, and apoptosis [112]. The role of LA for improving erectile dysfunction, benign prostatic hyperplasia, and hyperglycemic stroke in diabetic rats and mice, respectively, has been suggested in the existing literature [113,114,115]. Interestingly, the recent study of Kisioglu et al. (2024) [116] indicates that the oral intake of LA abrogates neuroinflammation, systemic inflammation, and anxiety-like behavior, and improves memory in a male C57BL/6 mice model of high-fat/high-fructose diet-triggered neuroinflammation and neurobehavioral deficit. And the study suggests that the downregulation of CD36 expression, which provokes a microglial neuro-inflammatory state, may be linked to the neuroprotective mechanism of LA, as suggested earlier in earlier studies [117,118]. However, in a seemingly contrasting investigation [44], a reduction in the LA level in VCO was associated with the improved lipid profile in high-fat diet-induced hypercholesterolemic mice. The oral consumption of repeatedly heated VCO was found to elevate blood pressure and inflammatory biomarkers in rats [119] confirming the deleterious effect of toxic products in repeatedly heated oils already established in the literature.

Regarding pharmaceutical formulations, a study investigated a nanogel combining chitosan, thiocolchicoside LA (CTLA) for anti-inflammatory, antimicrobial, antioxidant, and cytotoxic effects. CTLA demonstrates substantial anti-inflammatory and antioxidant activity, comparable to diclofenac sodium, and effectively inhibited Streptococcus mutans and Staphylococcus aureus [120]. Additionally, LA has garnered attention in food and pharmaceutical applications for its antimicrobial and antioxidant properties. Found abundantly in VCO, LA contributes to skin health by combating microbial growth and oxidative stress. These applications underscore LA’s versatility and efficacy in promoting skin health and cosmetic formulations.

## 4. Anticancer Effects of VCO and LA

Although radiation and chemotherapy are the mainstays of cancer treatment, they produce intractable side effects in patients [23]. The emergence of resistance to chemotherapeutic drugs in several cancer types is another formidable hurdle in oncotherapy [3]. Thus, there is a need for effective and safe bioprophylactics and biotherapeutics in cancer therapy. Natural VCO offers a myriad of health benefits, and, interestingly, the existing literature has shown the anticancer effects of VCO and LA.

### 4.1. Anticancer Effects of VCO

A growing number of published findings indicate the anticancer potential of VCO in breast, lung, colon, oral, and liver cancers (Table 1). In support of this, the in vitro study of Verma et al. (2019) [121], in which three different coconut oils, VCO, Processed Coconut Oil (PCO), and Fractionated Coconut Oil (FCO), were evaluated on the liver and oral cancer cell lines, demonstrates this effect. Although specific anticancer mechanisms were not explored, the study confirmed the anti-proliferative potentials of these coconut oils in the liver and oral cancer cell lines using an MTT cytotoxicity assay within 72 h of incubation. Remarkably, the oils show anticancer effects on the HepG2 and KB cell lines, leading to the conclusion of the authors that the VCO, PCO, and FCO have anticancer efficacy and may be used for the treatment of cancer, especially the liver and oral cancers. However, the anticancer efficacy may be dependent on the type of fatty acid profiles in the coconut oils [121]. The coconut oil nanoemulsion that contains the anticancer drug methotrexate (MTX) enhances the antiproliferative activity of MTX by a cytotoxic effect on A549 non-small-cell lung cancer cells with apoptotic morphological characteristics. The nanoemulsion inhibits A549 proliferation two times more than MTX alone. This suggests the anticancer role of VCO in the nanoemulsion [122]. Interestingly, in this study, the oxidative stress widely reported for the MTX-induced side effect toxicity was significantly ameliorated. Although a number of studies have reported that VCO is able to suppress the oxidative stress associated with chemotherapy toxicity [8,123], the double-edged benefit herein exhibited by the VCO-MTX nanoemulsion is significant. Coconut oil exerts moderate growth inhibition on the skin cancer cell line; however, the lack of a significant difference from normal melanocytes limits its clinical relevance and suggests a non-selective effect [124]. In an earlier study, coconut oil rich in LA has been reported to significantly inhibit the growth of HT-29 malignant human colon cells compared to linoleic acid [125]. A study investigated the impact of the lipid composition (saturated fats) of a high-fat diet on pro-oncogenic processes [97]. This study included diets supplemented with saturated-fatty-acid (SFA)-rich coconut oil against an experimental colon cancer model induced by azoxymethane/dextran sodium sulfate. Interestingly, the SFA-rich coconut oil diet is protective against the induced model of colon cancer via the mechanism of elevated colonic mucin 2 protein content involved in the physiological maintenance of intestinal barrier integrity. The VCO treatment induces reduced cell viability with the induction of apoptosis characterized by morphological alterations, including the appearance of massive cytoplasmic vacuolization and the blebbing of the cell membrane in the A549 and NCI-H1299 lung cancer cell lines [96]. Furthermore, the anticancer effects of coconut oil extracted by different methods were evaluated in human neuroblastoma cells (SH-SY5Y) cultured in vitro [7]. Ramya et al. (2022) [7] shows that VCO and crude coconut oil (ECO) provoke apoptotic characteristics consistent with morphological changes such as nuclear fragmentation, chromatin condensation, and the disintegration of membrane integrity in SH-SY5Y neuroblastoma cells. The VCO and ECO treatments exert greater growth inhibition and nuclear damage leading to apoptotic cell death than the refined coconut oil (RCO) treatment. The mitochondrial membrane potential (∆ψm) determination with JC-1 staining reveals that VCO significantly reduced the ∆ψm than ECO, indicating the mechanism underlying the cell growth inhibition and apoptosis in the study. A similar anticancer effect of VCO inhibits SkBr-3 breast cancer cell proliferation; it also synergizes with trastuzumab treatment to enhance the growth inhibitory effect in vitro [126]. The network pharmacology analysis of the major saturated fatty acids in VCO, like LA, caprylic acid, capric acid, and myristic acid against identifying the target proteins and pathways regulated by VCO, was investigated [89]. Through this computational analysis, 17 cancer-associated proteins and pathways involving various neoplasms were identified. The VCO-gene expression data implicated different neoplasms. However, the experimental validation of the in silico data reveals the anticancer effect of VCO on HepG2 liver cancer cells only (Table 3). It is important to note that the findings in the study of Pruseth et al. (2020) [89] are preliminary data and require further work.

### 4.2. Anticancer Effect of LA

Virgin coconut oil contains MCFAs, and LA is the most abundant MCFA in all coconut oil samples [7,40,76]. However, LA is not only present in VCO but also in palm kernel oil, fruits, seeds, and breast milk. Lauric acid is 45 g/100 g of the edible portion of coconut oil or palm kernel oil [127]. The literature shows the anticancer properties of LA and suggests it to be a very active agent for cancer treatment [127]. In vitro cell culture studies and findings exist in the published papers underscoring the anticancer mechanisms of LA in colorectal cancer, liver cancer, endometrial cancer, and human skin epidermoid carcinoma. Although there are a few studies currently, the insights from the available data consistently show that LA exposure imparts growth inhibitory effects on several cancer cell lines (Table 4).

To confirm the apoptosis-inducing effect of VCO, SH-SY5Y neuroblastoma cells were exposed to LA [7]. LA exhibits dose-dependent and time-dependent cytotoxic effects on the SH-SY5Y cells demonstrated by a marked growth inhibition. In addition, LA provokes an anticancer mechanism consistent with ROS-mediated apoptosis by reducing the mitochondrial membrane potential in SH-SY5Y cells. A reduction in the potential indicates mitochondrial dysfunction that may lead to low ATP production, which will impart a severe blow on the survival of cancer cells. VCO and crude coconut oil caused the release of ROS, key oxidative stress response genes, and inflammatory genes in SH-SY5Y cells. On this ground, unfolding the redox nexus of antioxidant Nrf2 and the NF-κB signaling pathway may be important in future studies in order to lay a foundation for the effect of VCO in this type of cancer. It is worth noting that the antiproliferative effects observed lacked validation against noncancerous cell lines which may reduce their translational significance. The human hepatocellular carcinoma cell line (HepG2), colorectal cancer (CRC) cell line (HCT-15), and murine macrophages (Raw 264.7) were used to investigate the effects of LA and other MCFAs [30]. Lauric acid demonstrates a dose-dependent inhibition of cell growth. In addition, the LA treatment at 30 and 50 μg/mL in HCT-15 reveals epidermal growth factor receptor (EGFR) downregulation and diminished EGFR levels in the lipid rafts, leading to morphological characteristics of apoptosis [30]. EGFR signaling is crucial to cancer cell survival and progression, and its alteration or downregulation is associated with cytotoxicity, the induction of apoptosis, a anti-metastatic effect, and cancer cell death [138,139]. Therefore, cetuximab, an EGFR inhibitor, is the main targeted chemotherapy against metastatic CRC, although the cetuximab chemotherapy is limited to a subtype of CRC patients without mutated BRAF or KRAS genes. This is because cetuximab chemotherapy is challenged with chemoresistance, and may even trigger uncontrolled cell proliferation, cell growth, and even metastases in CRC patients with a mutation of either BRAF or KRAS genes [140,141]. But, in the study of Weng et al. (2016) [128], it was found that LA could sensitize KRAS/BRAF-mutated CRC cells to cetuximab chemotherapy. The authors observed that LA orchestrates the sensitization to cetuximab via an elevated microRNA-378 expression accompanied by a decreased expression of mitogen-activated protein (MAP)/extracellular signal-regulated kinase-2 (ERK-2). It was also noted that LA could inhibit growth in KRAS/BRAF-mutated CRC cells even without cetuximab [128]. The finding is important for the possible synergistic anticancer effect of LA and shedding new light on overcoming the therapy resistance of the BRAF or KRAS mutation in CRC patients.

Moreover, the effects of LA were observed in a study that indicates the apoptosis induction potential of LA to kill colon cancer cells [129]. In this study, LA was able to induce ROS coupled with a reduced level of glutathione, which mechanistically provokes apoptotic alterations and cell cycle arrest at the G0/G1 and G2/M phases. However, the absence of control comparisons to a normal colon epithelium calls into question the therapeutic selectivity of this effect. Furthermore, LA was also found to inhibit the growth of HT-29 malignant human colon cell line [125]. A mechanistic study indicates that LA triggers a signaling pathway that leads to antiproliferative and pro-apoptotic effects in both endometrial (Ishikawa) and breast (SkBr3) cancer cells [130]. The LA-activated signaling and molecular mechanism stimulates ROS stress and the phosphorylation of c-Jun, ERK1/2, and EGFR, and the elevated expression of c-fos. The Rho-associated kinase was also phosphorylated and could be connected with ROS generation, which is a double-edged sword, activating and or inhibiting biological processes modulated by protein kinases, phosphatases, and several other enzymes [127]. Gemcitabine chemotherapy is the first-line therapy for the treatment of bladder cancer; however, due to chemoresistance linked to the depressed expression of cellular membrane human equilibrative nucleoside transporter 1 (hENT1), the facilitative transporter of gemcitabine (GEM), its use is limited in chemotherapeutic regimens. Although a number of fatty-acid-based GEM derivatives have been developed in order to enhance the transport of GEM into cancer cells [142], the LA-GEM derivative conjugate shows interesting findings against human bladder cancer [27]. The LA-based GEM conjugate (SZY-200) and LA alone were evaluated for anticancer effects. SZY-200 inhibits the proliferation of bladder cancer cells by inducing cell cycle arrest and apoptosis comparable to GEM alone and CP-4126 (an elaidic acid–GEM conjugate), but the interesting finding in this study is that SZY-200’s entry into bladder cancer cells does not rely on hENT1, a transporter protein that promotes GEM resistance. In addition, LA alone could inhibit the proliferation of bladder cancer cells, while SZY-200 downregulates the expressions of the peroxisome-proliferator-activated receptor gamma (PPAR-γ) and prostaglandin-endoperoxide synthase 2 (PTGS2) genes involved in the development of bladder cancer [27]. Clearly, the role of LA is evident in the by-passing of hENT1 for GEM accumulation in bladder cancer cells, triggering antiproliferation, cell cycle arrest, and apoptosis.

**Figure 4:**
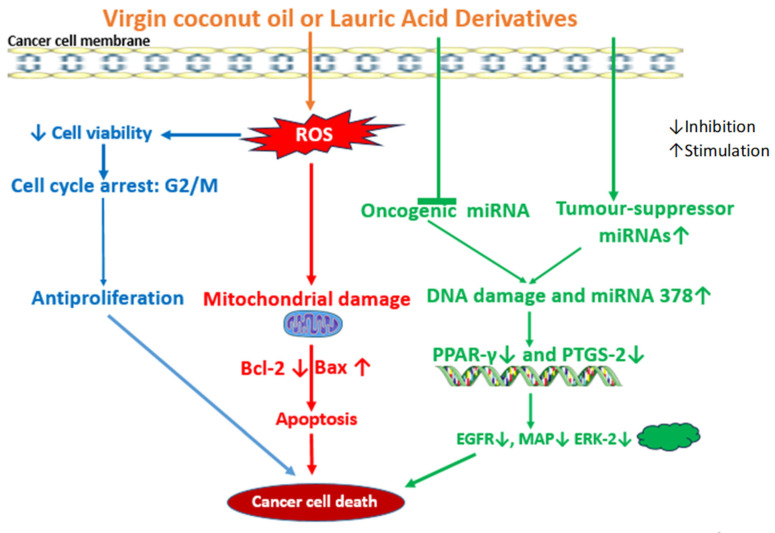
Molecular mechanisms associated with anticancer effects of VCO or LA and their derivatives. VCO, LA, and their derivatives, upon entering the cell, interact directly with molecules and organelles to generate ROS and/or oxidative stress, which leads to depressed cancer cell viability, anti-proliferation, mitochondrial damage, and modulation of cancer-cell-death-related miRNAs. These alterations cause apoptotic cascades via cell cycle arrest, depressed expression of proteins, and genes that provoke cancer cell death.

Another interesting study has indicated the influence of LA on microRNA expression in cancer cells. The LA treatment of KB-1 cells and HepG2 cells suppresses the expression of oncogenic microRNA and upregulates the expression of tumor-suppressor microRNAs [137]. These LA-modulated microRNAs are associated with cancer pathways with respect to apoptotic signaling, DNA damage, cell growth, cell cycle phase transition, cell response to stress, p53 mediation. protein signaling pathway, cyclin-dependent protein kinase activity, and epidermal growth factor receptor signaling pathways, according to the metabolic pathways analysis by the in silico GeneMANIA software. However, these associations remain hypothetical and require experimental validation to confirm any direct mechanistic link [137] (Figure 4). MicroRNAs are regulators of major cellular biological processes, including apoptosis, cell division and growth, tissue differentiation, and regeneration, and their dysregulation is a crucial molecular factor that contributes to the development of cancer [143]. For example, there is the upregulation of miR-17-92 and miR-21 in cancer, whereas microRNAs let-7 and miR-34 families are downregulated [137,144]. However, there is a paucity of data on the effect of LA on microRNA expression for the anticancer effect and mechanisms.

Hence, it is suggested that lauric acid exerts anticancer effects in various models mediated by distinct, context-dependent molecular pathways. The observed mechanisms—ranging from ROS-mediated apoptosis to EGFR downregulation and microRNA modulation—highlight the heterogeneity in cellular response, rather than a single universal mechanism.

#### LA-Based Nanocarriers for Therapeutic Delivery

A report has emerged on a role of LA in thermotherapy or hyperthermia—a technique of anticancer therapy that induces a hyperthermal stress at a temperature range of 41–45 °C in the tumor site. It is currently a widely used method for treating tumors, especially breast cancer without surgery [131]. The physical property of LA as an organic phase change material that absorbs and releases thermal energy during the process of melting (~43 °C) and freezing, respectively, is being explored. This makes LA applicable as a powerful chemotherapeutic agent in combination with thermotherapy. In support of this, a study synthesized novel nanocapsules consisting of an LA core encapsulated in a silica shell (SiO2@LA NPs). The LA-encapsulated nanomaterial was suitable for releasing hyperthermal stress on the breast cancer cells (MCF-7), disrupting the cell membrane and inducing oxidative stress, apoptosis, and morphometric alterations [131]. The study shows that thermal treatment alone did not cause a reduced viability of MCF-7 significantly but did with the LA nanoencapsulation in SiO2@LA NPs. It is widely published that VCO and LA have antibacterial and virucidal effects [56,71,145,146]. With this background, Xin et al. (2023) [132], thus, synthesized a nanocomplex conjugated to the platinum IV Pt(IV)-complex and LA to combat the Gram-negative bacteria, Fusobacterium nucleatum (Fn), that promotes chemoresistance in oxaliplatin chemotherapy against CRC. The bacteria also promote the proliferation and metastasis of CRC. In the study, the nanocomplex was encapsulated with hyaluronic acid to enhance biocompatibility and tumor targeting. Interestingly, the nanocomplex exhibits a notable anti-CRC activity via cytotoxicity mediated by ROS generation, and a high anti-Fn activity. Similar findings were found for LA-based nanodrug systems (5-FU-LA@PPL) which increased chemosensitivity, the combinational compatibility of 5-fluorouracil and oxaliplatin, and the suppressed tumor-resided Fn-associated CRC in the in vitro and in vivo colorectal cancer (HT-29) xenograft model [133]. LA was found to inhibit autophagy and reduce cell viability, demonstrating a higher cytotoxic effect compared with the drug 5-FU against Fn-infected CRC cells.

Considerable findings were obtained when MCFAs, including LA conjugated with 2-hydroxyanthraquinone, a derivative of the anticancer drug doxorubicin, were evaluated for an anticancer effect against breast cancer cell lines MDA-MB-231 and MCF-7, human colon cancer cell line HT-29 and Colo-205, and leukemia cell line K-562 [134]. Among others, the LA conjugate exerts anticancer effects on Colo-205 and MCF-7. According to the findings, the LA conjugate proved to be the most active against human colon and breast cancers among all the MCFA conjugates. The study concludes that the substitution of LA on the anthraquinone ring probably enhances lipophilicity, causing greater cell membrane permeation. In recent investigations, moreover, three LA-based hydrazones inhibit liver cancer cell HepG2 growth after 48 h of exposure in the MTT medium. The cytotoxic effect was ascribed to the LA–hydrazine moiety in the LA-based hydrazones [135]. A chitosan-based nanogel that incorporated LA (CTL) potentially inhibits oral cancer cell (KB-1) proliferation. The cytotoxic inhibition of the CTL nanogel was unfolded in the study and it involves CTL-nanogel-mediated cell cycle arrest and the modulation of the Bax/Bcl-2/caspase gene expression pro-apoptotic pathway [136]. It causes a reduced cell viability and apoptotic morphological changes in KB-1 cells via cell cycle arrest at the G2/M phase and ROS induction leading to increased gene expressions of Bax, Bad, and caspase-3, and tumor-suppressor genes p53 and p21, while the anti-apoptotic gene Bcl-2 exhibited decreased expression. The study provides supportive evidence to support the potential of the chitosan thiocolchicoside lauric acid nanogel as a potential therapeutic candidate for oral cancer treatment and possible application in cancer nanomedicine. While this study highlights one potential mechanism of action involving p21 in KB-1 oral cancer cells, further studies across different models are needed to confirm whether similar pathways are consistently involved.

## 5. VCO and LA: Protection Against Chemotherapy Toxicity

Traditional cytotoxic chemotherapy primarily targets rapidly proliferating cancer cells vulnerable to being killed by a cytotoxic agent, but, in reality, cytotoxic chemotherapy attacks non-targeted healthy cells due to its lack of specificity for tumor/cancer cells during treatment; therefore, the off-target attack constitutes a long-standing problem of the side effect toxicity in patients undergoing chemotherapy treatment [23,147]. This leads to a high systemic toxicity, and prevents the use of high drug doses that are required for theeffective killing of cancer cells, thus limiting the anticancer efficacy [28]. And oxidative stress, inflammation, and apoptotic mechanisms have been implicated principally to underlie the side effect toxicity mechanism of chemotherapy [3,23]. Notably, a growing body of literature has shown supportive evidence that natural products could mitigate the chemotherapy-induced side effect toxicity [3,23]. Apart from its anticancer properties, VCO has emerged as a functional food oil for mitigating the undesirable side effect toxicity associated with chemotherapy.

### 5.1. VCO Protects Against Chemotherapy Toxicity

**Figure 5:**
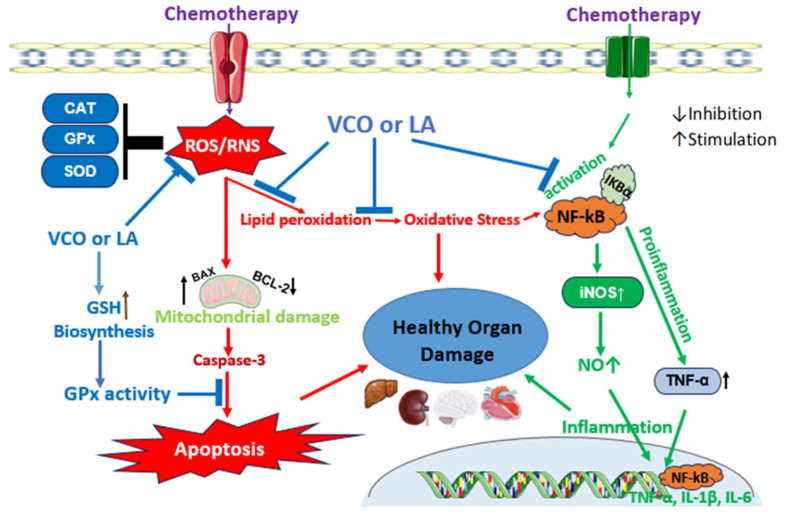
VCO or LA protective mechanisms against chemotherapy-induced organ toxicity. Chemotherapeutic drugs centrally induce ROS generation and oxidation stress for promotion of apoptosis and inflammatory mechanisms in healthy organs. VCO or LA inhibits these mechanisms at various key mechanistic points, leading to elevation of antioxidant mechanism and dampening or blockage of apoptosis and inflammatory process.

Due to the bioactive natural compounds in VCO, including caffeic acid, ferulic acid, vallinic acid, gallic acid, catechin, rutin, epicatechin, chlorogenic acid, hornavallin, p-coumaric acid, syringic acid, sinapic acid protocatechuic acid, and quercetin [40,45,148]. VCO is reported to combat the underlying toxicity mechanisms of chemotherapy. In male mice and female mice, the anticancer drug doxorubicin (DOX) induced neurobehavioral toxicity via the elevation of acetylcholinesterase (AchE), tumor necrosis factor-α (TNF-α), inducible nitric oxide synthase (iNOS), and altered neurobehavioral factors in both sexes [25]. The oral administration of VCO for 28 days was found to ameliorate some of the markers of neurotoxicity and neuroinflammation. VCO was evaluated for whether it can inhibit immunosuppression associated with DOX chemotherapy, because DOX could adversely modulate the interleukin (IL-2) and interferon-γ production, lymphocyte proliferation, NK cell cytotoxicity, and T lymphocyte CD4+/CD8+ ratio in tumor-bearing mice [149]. In the study, VCO was evaluated for its effect on CD4+ and CD8+ cells against DOX-induced immunosuppression in rats [150]. The CD cells, phagocytosis activity, lymphocyte proliferation, and capacity of macrophages were suppressed by a DOX injection. The DOX also caused a downregulation of IL-10, underscoring the initiation of inflammatory cascades in certain tissues. Interestingly, the oral VCO inhibits the immunosuppression and markedly enhances the CD4+, CD8+, and other immunological indices in the study [150]. It also inhibits the DOX-induced hepatic and cardiac damage and significantly reduces the activities of alanine aminotransferase (ALT), aspartate aminotransferase (AST), lactate dehydrogenase (LDH), and creatine kinase-MB in the serum of Wistar rats [151,152]. The lesions in the tissue histology were observed to be alleviated clearly in comparison to the non-treated DOX group. In a similar study, however, the oxidative inflammatory and apoptotic toxicity effects of DOX leading to liver toxicity were evidently shown. DOX induces significant increases in the serum levels of AST, ALT, and malondialdehyde (MDA), and a decrease in albumin coupled with elevated hepatic levels of pro-inflammatory NF-κB, TNF-α, and IL-6, and pro-apoptotic caspase-3 [24]. The liver levels of anti-apoptotic Bcl-2 as well as the antioxidant apparatus were shown to be considerably reduced in the study. VCO supplementation for 7 days was found to restore the liver function, antioxidant mechanism, and anti-apoptotic effect against the DOX toxicity mechanism in consonance with histological findings [24]. In a comparative investigation, hot-processed VCO (HPVCO) demonstrates a higher antioxidant potential than fermentation-processed VCO in a battery of in vitro antioxidant assays [153]. The authors validated this potential in an in vivo experimental model evaluating the antioxidant and nephroprotective effect of HPVCO and FPVCO against cisplatin (CP)-induced nephrotoxicity. CP is one of the foremost anticancer agents due to its wide efficacy in the treatment of various cancers [154,155]. However, the major side effect of CP chemotherapy is nephrotoxicity [154]. The results from the study show that CP provokes nephrotoxicity which was alleviated by HPVCO or FPVCO [153]. Notably, the HPVCO exerts the higher antioxidant restoration of hepatorenal function, and also inhibits myelosuppression and histological abrasions via the elevated activities of antioxidant enzymes and reduced glutathione. The study highlights the influence of processing methods on antioxidant bioactive compounds in VCO. This is similar to the findings against cyclophosphamide (CYP)-induced hepatorenal toxicity in a murine model. In the study, CYP induced the depression of the antioxidant status in the liver and kidney, followed by deleterious lipid peroxidative reactions and oxidative stress in mice [17]. However, the oral administration of VCO for 20 days alleviated the hematological alterations, lipid peroxidation, hepatorenal function, and oxidative stress. In the toxicity of CYP in lymphoid tissue, VCO prevents alterations in the hematological indices and histological changes both in the spleen and thymus organs [156]. Another anticancer agent of the class antimetabolites called methotrexate (MTX) is well-associated with organ toxicity via oxidative stress and inflammatory alterations. The nanoemulsion of VCO and MTX expressed depressed oxidative stress compared to the free MTX treatment in a model of experimental rats [122]. The VCO in the nanoemulsion was able to elevate the activities of antioxidant enzymes in the brain and lung tissues, while the tissue lipid peroxidation diminished significantly. VCO supplementation to rats in various studies has been shown to possess antioxidant and anti-inflammatory effects against MTX-induced hepatotoxicity [21,22] nephrotoxicity [21] neurotoxicity [8], and systemic toxicity [123]. In these studies, VCO exerts an effect that reverses oxidative stress and also triggers the mechanistic suppression of inflammatory cytokines in the delicate organs (Figure 5). Although there is a paucity of reports on the effect of VCO on the side effects of chemotherapy in cancer patients, the study of Law et al. (2014) [157] was able to show that VCO supplementation improves the quality of life among breast cancer patients in Kelantan, Malaysia. According to the human study, the side effect symptoms, including dyspnea, sleep difficulties, fatigue, and loss of appetite, body image, and sexual function, improved when compared to the patients in the control group. The study reports that there is still a deterioration in sexual enjoyment; the sample size of participants was not large though [157]. However, it is unknown, in this human study, whether VCO could ameliorate the side effect toxicity of the liver and kidney, which are very rampant following chemotherapy. Moreover, VCO reduces mucositis, a common side effect of cancer treatment, in nasopharyngeal carcinoma patients [158]. Bioactive polyphenols in VCO may be responsible for these observations, and more comprehensive studies are needed in order to investigate the anticancer activity and modulatory mechanisms of VCO against chemotherapy side effect toxicity (Table 5).

### 5.2. LA Protects Against Chemotherapy Toxicity

The effect of LA on the organ-specific side effect toxicity of chemotherapy is currently unknown in existing literature. However, cancer cachexia, characterized by weight and skeletal muscle loss, is an important adverse effect of anticancer chemotherapy, and there are no approved drugs for it [160]. In a mouse cachexia model, combining the oral administration of LA and glucose improves cancer-derived myocardial damage as demonstrated by the recovery of myocardial atrophy and MYL1—a marker of muscle maturity [159].

## 6. Conclusions and Future Perspectives

VCO and its most abundant saturated MCFA, LA, are natural products with numerous pharmacological applications for human health and industrial uses. VCO is obtained from mature coconut fruits using several traditional methods of extraction devoid of refining and deodorizing. The review of literature herein has revealed that VCO and LA are potential anticancer natural agents. Studies show that VCO, LA, and their synthesized nanocomplex conjugates and nanomaterials exert anticancer effects and also enhance the anticancer efficacy of standard chemotherapeutic agents. In cancer cells, they induce anti-proliferative effect via ROS generation and mitochondrial dysfunction, leading to Bax.Bcl-2/caspase apoptotic cell death, cell cycle arrest, EGFR/c-JUN/ERK1-2/c-fos altered pathway, and microRNA downregulation in various cancers. However, other cancer-related pathways, including ERK/JNK/MAPK, PI3K/Akt/mTOR, Wnt/β-catenin, JAK/STAT, and ferroptosis SLC7A11/GPX-4 axis, remain to be reported. The side effect toxicity of cancer chemotherapy is still an intractable challenge in oncotherapy. The literature reports indicate that VCO could suppress the toxicity of MTX, CYP, CP, and DOX chemotherapies by its antioxidant, anti-apoptotic, and anti-inflammatory activities in preclinical studies. There are no reports on the effect of VCO or LA on the toxicity of other chemotherapeutic agents, such as 5-fluorouracil, paclitaxel, docetaxel, tamoxifen, or topoisomerase inhibitors. It is significant that the effect of LA on chemotherapy toxicity is currently unclear in existing literature. Clinical trials exploring the effectiveness of VCO and LA in the adjuvant treatment and management of cancer are recommended.

As the therapeutic application of VCO and LA continues to expand, it becomes increasingly important to align safety evaluations with established regulatory frameworks. The current evidence mainly focuses on preclinical efficacy and mechanistic insights, while comprehensive safety assessments remain limited. Future research should adopt structured safety assessment protocols such as those outlined by the European Food Safety Authority (EFSA) and other international guidelines for food-related nanomaterials. Incorporating these assessments will be essential for ensuring the translational viability and public health relevance of VCO/LA-based nanoformulations in both functional foods and therapeutic contexts.

## Figures and Tables

**Table 1:** Bioactive components in VCO and their reported effects.

Bioactive Class	Bioactive Constituents	Reported Effect	References
Medium-chain fatty	LA (C12), capric acid (C10), caprylic acid (C8), caproic acid (C6)	Antimicrobial, immunomodulatory, antiviral	[40,44,45,46]
Polyphenol	Ferulic acid, catechins, p-coumaric acid,	Antioxidant, anti-inflammatory	[42]
Tocopherols	α-Tocopherol	Antioxidant	[45,47]
Phytosterols	β-Sitosterol	Hypocholesterolemia	[43]
Phenolic acids	Caffeic acid, gallic acid	Free radical scavenging, anti-inflammatory, blood glucose regulation, chemoprevention, and immunomodulation	[48,49]
Flavonoids	Quercetin	Antioxidant, vascular protection	[40]

**Table 2:** Compositional and metabolic overview of VCO, butter, and hydrogenated oils.

Component/Feature	VCO	Butter	Hydrogenated Oil
Medium-chain triglycerides	High	Low	Absent
Polyphenols	Moderate	Low	Absent
Trans-fats	Absent	Absent	High
Cholesterol	Absent	High	Variable
Absorption rate	Fast	Slow	Slow
Metabolic oxidation	Fast	Slow	Slow
LDL/HDL effect	Increases HDL, decreases LDL/HDL ratio	Increase LDL	Highly increase LDL

**Table 3:** Studies on anticancer effects of VCO and its derivatives.

VCO Nature	Cancer Type	Model/Cell Line	Effect/Activity	Dose	Timing	References
VCO	Liver and oral cancers	HepG2, KB	Antiproliferation	50–100 µg/mL	72 h	[121]
VCO nanoemulsion	Lung cancer	A549, EAC	Antiproliferation	Not specified	48 h	[122]
VCO	Skin cancer	Melanoma cell line	Antiproliferation	10–300 µg/mL	48 h	[124]
VCO	Colon cancer	Colon adenocarcinoma cell line	Antiproliferation	30 µg/mL	72 h	[125]
CO	Colon cancer	In vivo	↑ Colonic mucin 2 protein	High-fat SFA-rich diet	28 days	[97]
VCO	Lung cancer	NCI-H1299, A549	Antiproliferation ↑ Apoptosis	25–100 µg/mL	24–72 h	[96]
CO	Neuroblastoma	SH-SY5Y	Antiproliferation ↑ Apoptosis Mitochondrial damage	50–150 µg/mL	24–72 h	[7]
VCO-trastuzumab	Breast cancer	SKBR-3	Antiproliferation	Not specified	48 h	[126]

↑ = Stimulation.

**Table 4:** Studies on the anticancer effect of LA and its derivatives.

LA/Derivative	Cancer Type	Model/Cell Line	Effect/Activity	Dose	Timing	Normal Cell Control Used	References
LA	Neuroblastoma	ISH-SY5Y	↑ ROS, apoptosis, mitochondrial damage	20–100 µg/mL	24–72 h	No	[7]
LA	Liver and colorectal cancers	HepG2, HT-29	Antiproliferation, ↓ epidermal growth factor receptor (EGFR), and ↑ apoptosis, anti-metastatic effect	30–50 µg/mL	24–48 h	No	[30]
LA-cetuximab or LA alone	KRAS/BRAF-mutated colorectal cancer	HCT116	Antiproliferation, ↑ microRNA-378, ↓ MAP, ↓ ERK-2	25 µM	48 h	No	[128]
LA	Colon cancer	HCT116	Antiproliferation, ↑ ROS, ↓ GSH, ↑ apoptosis, G2/M cell cycle arrest	20–60 µM	24–48 h	Yes	[129]
LA	Endometrial and breast cancers	Ishikawa, MCF-7	Antiproliferation, ↑ apoptosis, ↑ EGFR/ERK	25–100 µM	24–48 h	Yes	[130]
LA	Colon cancer	HT-29	Antiproliferation	30 µM	72 h	Yes	[125]
LA-Gemcitabine or LA alone	Bladder cancer	T24	Antiproliferation, ↑ apoptosis, ↓ PPARG and ↓ COX2 genes, cell cycle arrest	10–40 µM	48 h	No	[27]
LA nanocapsule (SiO2@LA)	Breast cancer	MCF-7	↓ Cell viability, ↑ ROS, ↑ apoptosis	Not specified	24 h	Yes	[131]
LA-platinum nanocomplex	Colorectal cancer	HT-29	↓ Cell viability, ↑ ROS	5 µM	24 h	No	[132]
LA-nanodrug systems (5-FU-LA@PPL)	Colorectal cancer	Xenograft (BALB/c nude mice)	↓ Cell viability ↓ autophagy	5 µM	48 h	No	[133]
LA-conjugated 2-hydroxyanthraquinone	Breast, colon and leukemia cancers	HL-60, HCT116,	↓ Cell viability	10–25 µM	24–48 h	No	[134]
LA-based hydrazones	Liver cancer	HepG2	Antiproliferation	50 µM	48 h	No	[135]
LA-chitosan-based nanogel	Oral cancer	Ga9-22	Antiproliferation, ↓ cell viability, ↑ ROS, G2/M, cell cycle arrest, and ↑ Bad/Bax/p53 apoptosis	20 µg/mL	24 h	Yes	[136]
LA	Oral and liver cancers	HepG2, SCC-15	↓ Oncogenic microRNA, ↑ tumor-suppressor microRNA, cell cycle arrest, ↑ apoptosis	20 µg/mL	24–48 h	No	[137]

↑ = Stimulation. ↓ = Inhibition.

**Table 5:** Protective effects/mechanisms of VCO or LA against chemotherapy toxicity.

VCO or LA	Anticancer Drug	Type of Toxicity	Animal Model Details	Mechanistic Outcomes (% Improvement)	Dose and Timing	References
VCO	Doxorubicin (DOX)	Neurobehavioral toxicity	Swiss albino mice, DOX-induced, post-treatment, behavior assays	↓ AchE, TNF-α, iNOS; improved behavior; ↓ oxidative stress (approx. 35–50% change in markers)	5 mL/kg/day VCO × 28 days, DOX 5 mg/kg i.p.	[25]
VCO	Doxorubicin (DOX)	Immunosuppression	Balb/c mice, DOX-induced, pre-treatment, immune assays	↑ CD4+, CD8+, lymphocyte proliferation, macrophage activity (up to 45%)	10 mL/kg/day VCO × 14 days, DOX 10 mg/kg i.p.	[150]
VCO	Doxorubicin (DOX)	Hepatic and cardiac toxicity	Wistar rats, DOX-induced, post-treatment, histopathological evaluation	↓ ALT, AST, LDH, CK-MB (~30–40%); restored histology	5 mL/kg/day VCO × 21 days, DOX 5 mg/kg i.p.	[151,152]
VCO	Doxorubicin (DOX)	Liver toxicity	Wistar rats, DOX-induced, post-treatment, cytokine, and antioxidant evaluation	↓ AST, ALT, MDA, inflammatory cytokines (30–60%), ↑ Bcl-2, antioxidant enzymes	5 mL/kg/day VCO × 21 days, DOX 5 mg/kg i.p.	[24]
VCO	Cisplatin (CP)	Nephrotoxicity	Wistar rats, CP-induced nephrotoxicity, comparison of hot/cold VCO	↓ Creatinine, urea, ↑ SOD, CAT; higher in hot-processed VCO (20–50% difference vs. CP-only)	5 mL/kg/day VCO × 10 days, CP 5 mg/kg i.p.	[153]
VCO	Cyclophosphamide (CYP)	Hepatorenal toxicity	Swiss albino mice, CYP-induced, pre-treatment, hepatorenal function assessed	↑ GSH, ↓ MDA, restored organ function (30–45%)	10 mL/kg/day VCO × 20 days, CYP 200 mg/kg i.p.	[17]
VCO	Cyclophosphamide (CYP)	Lymphoid toxicity (altered hematological indices, histological spleen/thymus changes)	Swiss mice, CYP-induced, spleen/thymus histology, hematological changes	Improved spleen/thymus histology and hematology (qualitative only)	5 mL/kg/day VCO × 10 days, CYP 100 mg/kg	[156]
VCO	Methotrexate (MTX)	Oxidative stress & inflammation	Wistar rats, MTX-induced, pre-treatment with VCO nanoemulsion, antioxidant evaluation	↑ SOD, CAT, ↓ lipid peroxidation (35–60%)	5 mL/kg/day VCO nanoemulsion × 7 days, MTX 20 mg/kg	[122]
	Methotrexate (MTX)	Hepatotoxicity, nephrotoxicity, neurotoxicity, systemic toxicity	Wistar rats, MTX-induced, systemic toxicity markers assessed	↓ ALT, creatinine, TNF-α, IL-6; ↑ SOD, CAT (~40–60%)	5 mL/kg/day VCO × 10 days, MTX 20 mg/kg	[8,21,22,123]
LA	LA and glucose	Cachexia (muscle atrophy, weight loss)	C57BL/6 mice, cancer cachexia model, LA + glucose supplementation	↑ Muscle mass, ↑ MYL1 gene (approx. 30%)	250 mg/kg LA + 1 g/kg glucose × 14 days	[159]

↑ = Stimulation. ↓ = Inhibition.

## Data Availability

No new data were created or analyzed in this study.
